# Internet use by older adults with bipolar disorder: international survey results

**DOI:** 10.1186/s40345-018-0127-7

**Published:** 2018-09-04

**Authors:** Rita Bauer, Tasha Glenn, Sergio Strejilevich, Jörn Conell, Martin Alda, Raffaella Ardau, Bernhard T. Baune, Michael Berk, Yuly Bersudsky, Amy Bilderbeck, Alberto Bocchetta, Angela M. Paredes Castro, Eric Y. W. Cheung, Caterina Chillotti, Sabine Choppin, Alessandro Cuomo, Maria Del Zompo, Rodrigo Dias, Seetal Dodd, Anne Duffy, Bruno Etain, Andrea Fagiolini, Miryam Fernández Hernandez, Julie Garnham, John Geddes, Jonas Gildebro, Michael J. Gitlin, Ana Gonzalez-Pinto, Guy M. Goodwin, Paul Grof, Hirohiko Harima, Stefanie Hassel, Chantal Henry, Diego Hidalgo-Mazzei, Anne Hvenegaard Lund, Vaisnvy Kapur, Girish Kunigiri, Beny Lafer, Erik R. Larsen, Ute Lewitzka, Rasmus W. Licht, Blazej Misiak, Patryk Piotrowski, Ângela Miranda-Scippa, Scott Monteith, Rodrigo Munoz, Takako Nakanotani, René E. Nielsen, Claire O’Donovan, Yasushi Okamura, Yamima Osher, Andreas Reif, Philipp Ritter, Janusz K. Rybakowski, Kemal Sagduyu, Brett Sawchuk, Elon Schwartz, Claire Slaney, Ahmad H. Sulaiman, Kirsi Suominen, Aleksandra Suwalska, Peter Tam, Yoshitaka Tatebayashi, Leonardo Tondo, Julia Veeh, Eduard Vieta, Maj Vinberg, Biju Viswanath, Mark Zetin, Peter C. Whybrow, Michael Bauer

**Affiliations:** 1 Department of Psychiatry and Psychotherapy, University Hospital Carl Gustav Carus, Technische Universität Dresden, Dresden, Germany; 2 ChronoRecord Association, Fullerton, CA USA; 3 Bipolar Disorder Program, Neuroscience Institute, Favaloro University, Buenos Aires, Argentina; 4 AMEOS Klinika Holstein, Neustadt, Germany; 5 Department of Psychiatry, Dalhousie University, Halifax, NS Canada; 6 Unit of Clinical Pharmacology, University Hospital of Cagliari, Cagliari, Italy; 7 Discipline of Psychiatry, School of Medicine, University of Adelaide, Adelaide, SA Australia; 8 School of Medicine, IMPACT Strategic Research Centre, Deakin University, Geelong, VIC Australia; 9 University Hospital Geelong, Barwon Health, Geelong, VIC Australia; 10 Department of Psychiatry, The University of Melbourne, Parkville, VIC Australia; 11 Florey Institute of Neuroscience and Mental Health, Parkville, VIC Australia; 12 Orygen Youth Health Research Centre and the National Centre of Excellence in Youth Mental Health, Parkville, VIC Australia; 13 Department of Psychiatry, Faculty of Health Sciences, Ben Gurion University of the Negev; Beer Sheva Mental Health Center, Beer Sheva, Israel; 14 Department of Psychiatry, University of Oxford, Warneford Hospital, Oxford, UK; 15 Section of Neurosciences and Clinical Pharmacology, Department of Biomedical Sciences, University of Cagliari, Cagliari, Sardinia Italy; 16 Department of General Adult Psychiatry, Castle Peak Hospital, Hong Kong, China; 17 AP–HP, Hôpitaux Universitaires Henri-Mondor, Créteil, France; 18 Department of Molecular Medicine and Department of Mental Health (DAI), University of Siena and University of Siena Medical Center (AOUS), Siena, Italy; 19 Bipolar Disorder Research Program, Department of Psychiatry, University of São Paulo Medical School, São Paulo, Brazil; 20 Department of Psychiatry, University of Calgary, Calgary, Canada; 21 AP–HP, Hôpitaux Universitaires Henri-Mondor, INSERM U955 (IMRB), Université Paris Est, Créteil, France; 22 Department of Psychiatry, University Hospital of Alava, University of the Basque Country, CIBERSAM, Vitoria, Spain; 23 Department of Affective Disorders, Q, Mood Disorders Research Unit, Aarhus University Hospital, Aarhus, Denmark; 24 Department of Psychiatry and Biobehavioral Sciences, Semel Institute for Neuroscience and Human Behavior, University of California Los Angeles (UCLA), Los Angeles, CA USA; 25 Mood Disorders Center of Ottawa, Ottawa, Canada; 26 Department of Psychiatry, University of Toronto, Toronto, ON Canada; 27 Department of Psychiatry, Tokyo Metropolitan Matsuzawa Hospital, Setagaya, Tokyo Japan; 28 Department of Psychiatry, Cumming School of Medicine, University of Calgary, Calgary, Canada; 29 Unité Perception et Mémoire, Institut Pasteur, F-75015 Paris, France; 30 Bipolar Disorders Program, Hospital Clinic, University of Barcelona, IDIBAPS, CIBERSAM, Barcelona, Catalonia Spain; 31 Department of Clinical Psychology, NIMHANS, Bangalore, 560029 India; 32 Leicestershire Partnership NHS Trust, Leicester, UK; 33 Institute of Clinical Research, Research Unit of Psychiatry, University of Southern Denmark, Odense, Denmark; 34 Department of Psychiatry, Psychiatry in the Region of Southern Denmark, Odense, Denmark; 35 Aalborg University Hospital, Psychiatry, Aalborg, Denmark; 36 Department of Clinical Medicine, Aalborg University, Aalborg, Denmark; 37 Department of Psychiatry, Wroclaw Medical University, Wroclaw, Poland; 38 Department of Neuroscience and Mental Health, Federal University of Bahia, Salvador, Brazil; 39 Michigan State University College of Human Medicine, Traverse City Campus, Traverse City, MI USA; 40 Department of Psychiatry, University of California San Diego, San Diego, CA USA; 41 Affective Disorders Research Project, Tokyo Metropolitan Institute of Medical Science, Setagaya, Tokyo Japan; 42 Department of Psychiatry, Psychosomatic Medicine and Psychotherapy, University Hospital Frankfurt, Goethe-University Frankfurt am Main, Frankfurt, Germany; 43 Department of Adult Psychiatry, Poznan University of Medical Sciences, Poznan, Poland; 44 Department of Psychiatry, University of Missouri Kansas City School of Medicine, Kansas City, MO USA; 45 Croton on Hudson, NY USA; 46 Department of Psychological Medicine, Faculty of Medicine, University of Malaya, Kuala Lumpur, Malaysia; 47 City of Helsinki, Department of Social Services and Health Care, Psychiatry, Helsinki, Finland; 48 Department of Psychiatry, Department of Medicine, University of Hong Kong, Hong Kong, China; 49 McLean Hospital and Harvard Medical School, Boston, MA USA; 50 Lucio Bini Center, Cagliari, Rome, Italy; 51 Psychiatric Center Copenhagen, Copenhagen, Denmark; 52 Department of Psychiatry, NIMHANS, Bangalore, 560029 India; 53 Department of Psychology, Chapman University, Orange, CA USA

## Abstract

**Background:**

The world population is aging and the number of older adults with bipolar disorder is increasing. Digital technologies are viewed as a framework to improve care of older adults with bipolar disorder. This analysis quantifies Internet use by older adults with bipolar disorder as part of a larger survey project about information seeking.

**Methods:**

A paper-based survey about information seeking by patients with bipolar disorder was developed and translated into 12 languages. The survey was anonymous and completed between March 2014 and January 2016 by 1222 patients in 17 countries. All patients were diagnosed by a psychiatrist. General estimating equations were used to account for correlated data.

**Results:**

Overall, 47% of older adults (age 60 years or older) used the Internet versus 87% of younger adults (less than 60 years). More education and having symptoms that interfered with regular activities increased the odds of using the Internet, while being age 60 years or older decreased the odds. Data from 187 older adults and 1021 younger adults were included in the analysis excluding missing values.

**Conclusions:**

Older adults with bipolar disorder use the Internet much less frequently than younger adults. Many older adults do not use the Internet, and technology tools are suitable for some but not all older adults. As more health services are only available online, and more digital tools are developed, there is concern about growing health disparities based on age. Mental health experts should participate in determining the appropriate role for digital tools for older adults with bipolar disorder.

## Background

The world’s population is living longer, with the percentage of people over age 60 years expected to nearly double from 12 to 22% between 2015 and 2050 (WHO 2015). Today, up to 25% of the population with bipolar disorder is age 60 years or older (Sajatovic et al. 2015). Older adults with bipolar disorder differ in the disease onset and clinical course, and most have multiple medical comorbidities especially endocrine, respiratory and cardiovascular conditions (Lala and Sajatovic 2012). Digital technology provides a framework to improve care for older adults with bipolar disorder by enabling remote visits, online psychological interventions, health monitoring, information seeking, peer support groups and self-management tools (Gliddon et al. 2017; Hidalgo-Mazzei et al. 2015; Torous et al. 2016).

In addition to providing help with bipolar disorder, Internet use by older adults in the community may decrease loneliness, and increase social support (Forsman and Nordmyr 2017; Heo et al. 2015). Internet use may also contribute to maintaining health literacy, or the ability to read, understand and act on health information (Kobayashi et al. 2015; Andrus and Roth 2002). Government and health care providers increasingly use the Internet as the primary form of communication about health and social services (Chang et al. 2015). Digital technologies are viewed as a means to maximize independence and facilitate aging in place, including for those with disabilities (Agree 2014; Reeder et al. 2013; Schulz et al. 2015), and to provide cost-effective care for the growing elderly population (Deloitte 2015). Most studies of Internet use involve community dwelling older adults and do not focus on mental illness. As the role of online services and monitoring technologies increases, more understanding of Internet use by older adults with bipolar disorder is needed.

To gain insight into online information seeking by patients with bipolar disorder, we previously surveyed 1222 adult outpatients with bipolar disorder living in 17 countries between March 2014 and January 2016 (Bauer et al. 2016; Conell et al. 2016). Of the patients in the survey, 81% used the Internet, a percentage similar to that of the general public (Bauer et al. 2016). The purpose of this analysis was to compare Internet use between the older adults, defined as 60 years or older, and younger adults less than 60 years, who completed this survey.

## Methods

The 39-question survey was anonymous, and took about 20 min to complete. The survey was paper based to maximize participation including of those who do not use the Internet. All participants were recruited locally by their psychiatrist with no online recruitment. The study was approved by institutional review boards according to local requirements. The patients who completed the survey resided in 17 countries. The survey was translated into 12 local languages: Chinese, Danish, Finnish, French, German, Hebrew, Italian, Japanese, Polish, Portuguese, Spanish, and English (versions for US/Canada, UK and Australia). The 1222 surveys were received from patients in Australia (N = 22), Brazil (N = 100), Canada (N = 109), Denmark (N = 209), Finland (N = 16), France (N = 50), Germany (N = 82), Hong Kong (N = 91), India (N = 30), Israel (N = 46), Italy (N = 80), Japan (N = 35), Malaysia (N = 25), Poland (N = 125), Spain (N = 82), UK (N = 50), and the US (N = 70).

The survey questions and methodology were published previously (Bauer et al. 2016; Bauer R et al. 2017; Conell et al. 2016). Since paper-based surveys were used, duplicate data entry was performed to minimize data entry errors. A model to evaluate the differences in Internet use by those age 60 years and older was estimated using the generalized estimating equation (GEE) statistical technique to accommodate imbalances in the number of responses from collection sites, and correlation in survey responses within collection sites. Variables significant at the 0.05 level in univariate analyses were included in the multivariate model estimates. SPSS version 24.0 was used for all analyses.

## Results

1222 patients completed the survey. The patients were 62% female, had a mean age of 44 years (SD 13.8) ranging between 17 and 86 years, and completed 14 (SD 3.2) years of education. The demographic characteristics are shown in Table 1. Of the 1222 patients, 81% used the Internet (976 of 1212 valid responses) (Bauer et al. 2016).

**Table 1:** Patient demographics (N = 1222)^a^

	Age 60 or older (N = 187)		Age 59 or younger (N = 1021)		All ages (N = 1208)	
	N	%	N	%	N	%
Diagnosis						
BP I	107	58	657	65	764	63
BP II	70	38	308	30	378	32
BP NOS	8	4	48	5	56	5
Gender						
Female	120	64	637	62	757	62
Male	68	36	390	38	458	38
Employment status						
Full-time	31	17	529	52	560	47
Not full-time	156	83	482	48	638	53
Marital status						
Married or living with partner	112	60	478	47	590	49
Not married	76	40	543	53	619	51
Income group						
Upper income	14	8	66	7	80	7
Middle income	105	57	487	48	592	49
Lower income	65	35	468	45	533	44
Live alone						
Yes	54	29	245	24	299	25
No	131	71	777	76	908	75
Mood in last 6 months						
Mostly normal	118	63	460	45	578	48
Mostly not normal	69	37	561	55	630	52
BP disorder interfered with regular activities						
Frequently or sometimes	89	47	676	66	765	63
Rarely or never	99	53	347	34	446	37
Confident managing living						
Very confident	89	48	363	36	452	38
Not very confident	98	52	659	64	754	62
Confident knowing when to see physician						
Very confident	117	62	578	57	695	57
Not very confident	71	38	445	43	516	43

	N	Mean (SD)	N	Mean (SD)	N	Mean (SD)
Years of education	184	13 (3.6)	1010	14 (3.1)	1194	14 (3.2)
Age of onset	186	36 (13.6)	1011	25 (9.4)	1197	27 (10.9)

^a^14 patients were missing responses to questions on age or “Do you use the Internet”? All missing values were excluded

There were 1208 valid responses to both the questions on age and “Do you use the Internet?” Of the 1208 patients, 187 were 60 years or older and of these 88 (47%) used the Internet. Of the 1021 younger adults, 884 (87%) used the Internet. Table 2 shows the best fitting model to assess differences in Internet use between the older adults and younger adults. The model includes variables for age 60 years or older, years of education, and if bipolar disorder sometimes or frequently interfered with regular activities. The estimated coefficients suggest that if age was 60 years or older, the odds of using the Internet will decrease by 86%, a 1 year increase in education will increase the odds of using the Internet by 30%, and if bipolar disorder interferes with regular activities, the odds of using the Internet will increase by 76%.

**Table 2:** Explanatory model based on responses from the patients who use the Internet (N = 1208)

Independent variables^a^			
Parameter	Significance	OR	95% CI
Intercept	< 0.001	0.136	0.053, 0.349
Age 60 years or older^b^	< 0.001	0.141	0.082, 0.241
Bipolar disorder sometimes or frequently interferes with regular activities	0.001	1.764	1.246, 2.497
Years of education	< 0.001	1.302	1.229, 1.380

^a^Patients with missing values were not included

^b^187 patients were age 60 years or older at time of study

Of those who used the Internet, 689/880 (78%) of younger adults and 59/88 (67%) of older adults looked for information on bipolar disorder. While this appears similar, insufficient data were available on the older adults for a more detailed statistical analysis.

## Discussion

Older adults with bipolar disorder used the Internet much less frequently than younger adults. Overall, 47% of older adults used the Internet versus 87% of younger adults. This finding is consistent with prior research on older adults not specific to bipolar disorder. Internet use by community dwelling older adults is increasing, with studies reporting percentages between 36 and 67%, but remains considerably lower than for younger adults (Levine et al. 2016; Friemel 2016; Yu et al. 2016; Anderson and Perrin 2017; Chang et al. 2015). As in prior research, more education and experiencing symptoms were associated with increased Internet use (Yu et al. 2016; Gell et al. 2015; Powell and Clarke 2006; Gallagher and Doherty 2009; Flynn et al. 2006), although symptoms of depression, and cognitive decline may decrease use in older adults (Choi and Dinitto 2013; Levine et al. 2018). Smartphone use by community dwelling older adults is even lower than Internet use, at about 40% (Anderson and Perrin 2017). In this survey, considering Internet users of all ages, 89% accessed the Internet to find information about bipolar disorder from a computer compared with 11% from a smartphone or tablet (Conell et al. 2016).

Older adults are diverse, differing in age, education, income, living situation, employment, and experience with technology. Notably, in a US survey of 567 adults age 60 or older, those who used the Internet were often comfortable doing so, and may have a job requiring computer use (Chang et al. 2015). In an international survey, information technology professionals over age 50 experienced less trouble working with multiple devices than younger workers (Patrizio 2016). However, as in this study, many older adults with or without bipolar disorder do not use the Internet. The reasons are complex and include difficulty learning technical skills, high costs of computers, mobile devices and broadband services, attitudes towards technology, increasing age, language issues for immigrants, cognitive decline, preference for traditional media, low health literacy, and relocation to a nursing home (Kuerbis et al. 2017; Fischer et al. 2014; Levine et al. 2018; Levy et al. 2015; Nimrod 2017; Chang et al. 2015). Some older adults are concerned that technology use will reduce face-to-face interactions, including contact with health care providers, and increase isolation (Kang et al. 2010; Kuerbis et al. 2017).

Regardless of Internet use, older adults view health care professionals as the primary and most trusted source of information (Hall et al. 2015; Medlock et al. 2015). Patients of all ages would prefer to learn about a serious mental illness by direct conversation with their psychiatrist (Hallett et al. 2013). Some older adults do not trust the Internet as a source of health information (Sbaffi and Rowley 2017; Zulman et al. 2011). In studies of patients with a mean age ≥ 50 years, those with a strong therapeutic relationship with a physician were less likely to search for health information on the Internet (Hou and Shim 2010), and more likely to defer decision making to the physician (Park et al. 2014). In this survey, the primary reason why Internet users of all ages did not seek information about bipolar disorder was because they prefer to rely on information from a physician (Bauer et al. 2016).

Regardless of age, most patients who did not use the Internet in this survey lacked technical skills (Bauer et al. 2016). One option to increase technology use by older adults is to provide training, but there are many serious concerns with novice Internet users. The elderly are such frequent targets for financial fraud that it is considered a public health problem in the US (Burnes et al. 2017; CDC 2015), and the scams targeting older adults have moved online (FBI 2014; Carlson 2007). In 2016, in the US, adults over age 60 were the largest group of victims of Internet crime, and suffered the largest monetary losses (FBI 2016). Factors that increase vulnerability to online fraud include low technical skills, individual traits, cognitive impairment, and depression in older adults (Monteith and Glenn 2016; Lichtenberg et al. 2016). Many older adults are not knowledgeable about Internet security hazards and measures to protect privacy (Grimes et al. 2010; Home Instead 2017; Holtfreter et al. 2015; White et al. 2017). Furthermore, many people of all ages have little understanding of privacy issues related to digital technology. For example, in this survey, 43% of patients of all ages searched the Internet for information about bipolar disorder because they mistakenly thought they were anonymous online (Conell et al. 2016).

In addition to financial fraud, older adults may fall victim to risky online medical activities. Older adults who are seeking to save money by purchasing expensive prescription drugs online will primarily be presented with rogue pharmacies that do not require a prescription (Monteith and Glenn 2017; Monteith et al. 2016). Some of the risks of using rogue pharmacies include counterfeit drugs, low-quality drugs, unapproved drugs, substitutions of strengths and formulations, drug interactions, adverse reactions, and financial fraud (Mackey and Nayyar 2016; Mackey and Liang 2011; GAO 2014). Another problem area involves the online advertising of unnecessary or inappropriate medical screening tests that are not included in evidence-based guidelines (Lovett et al. 2012; Lovett and Mackey 2013).

Older adults with physical limitations use the Internet less frequently than healthier older adults (Gell et al. 2015; Levine et al. 2018). Many older adults have vision, hearing, and dexterity impairments. Assistive technologies offer innovative options to get connected such as low-vision software for oversized monitors, speech amplification phones using landlines, and tremor stabilizing mouse controls (BT 2013; Watanabe et al. 2015; Fischer et al. 2014). More emphasis is needed on finding the optimal individualized approach for older adults to use digital technology rather than focusing on standard mobile devices (Fischer et al. 2014; Kuerbis et al. 2017). Additionally, technology approaches that combine data from those with and without Internet access, such as interactive voice response (IVR), should be considered for projects involving older adults (Verma et al. 2014; Piette et al. 2013).

There are some limitations to this report. The survey was not designed to study technology habits of older adults. The study participants do not reflect the demographic composition of the countries. People with bipolar disorder who did not seek professional help did not participate. People who did not understand the local language may not have participated. All data were self-reported and there was no follow-up discussion of responses. Many issues related to digital technology use by older adults were not discussed. These include the complex ethical challenges, quality of web sites and validity of digital tools for bipolar disorder (Bauer M et al. 2017), physiological effects of blue light exposure from digital devices (Bauer et al. 2018), and the potential for digital assistive tools to erode skills, decrease motivation, and promote a false sense of security (Schulz et al. 2015).

It is important to remember that technology will keep evolving (Arthur 2010). There will be disparities in the adaption of the new products and services, leaving digital equality a continuously moving target (Hilbert 2014, 2016). Young adults of today who are very comfortable using smartphones will continue to use smartphones as they age, and struggle with the new technologies available when they are seniors. The need to respect generational differences in the preferred means to access health information, minimize the burden of new technologies on older adults, and implement programs that expand access and lessen the negative impacts of digital inequalities will remain in the future.

In conclusion, the finding that many older adults with bipolar disorder do not use the Internet confirms the need for further investigation of technology habits, the efficacy of digital tools, and how best to determine who will use these tools appropriately and safely. Today, technology based tools and treatments are suitable for some but not all older adults with bipolar disorder. With government and health care providers increasingly relying on electronic communication, it is important to remember that many older adults do not use the Internet. As the population is aging and more health services are only available online, there is concern about growing health disparities for older adults with bipolar disorder. Mental health experts should contribute to defining the appropriate role for technologies in the care of older adults with bipolar disorder.
